# *CheckMyMetal*: a macromolecular metal-binding validation tool

**DOI:** 10.1107/S2059798317001061

**Published:** 2017-02-22

**Authors:** Heping Zheng, David R. Cooper, Przemyslaw J. Porebski, Ivan G. Shabalin, Katarzyna B. Handing, Wladek Minor

**Affiliations:** a Molecular Physiology and Biological Physics, University of Virginia, Charlottesville, VA 22908, USA

**Keywords:** metal-binding environment, validation, coordination geometry, *CheckMyMetal*

## Abstract

Metals are essential in many biological processes, and metal ions are modeled in roughly 40% of the macromolecular structures in the Protein Data Bank (PDB). However, a significant fraction of these structures contain poorly modeled metal-binding sites. *CheckMyMetal* (*CMM*) is an easy-to-use metal-binding site validation server for macromolecules that is freely available at http://csgid.org/csgid/metal_sites. The *CMM* server can detect incorrect metal assignments as well as geometrical and other irregularities in the metal-binding sites. Guidelines for metal-site modeling and validation in macromolecules are illustrated by several practical examples grouped by the type of metal. These examples show *CMM* users (and crystallographers in general) problems they may encounter during the modeling of a specific metal ion.

## Abstract 2

The metal-site validation tool *CheckMyMetal* is described, with examples to follow.

## 1. Introduction   

Metals are present in many macromolecules, and they are often essential to maintain the structural stability of the macromolecule. For example, magnesium has been shown to maintain the stability of many RNA structural motifs (Zheng *et al.*, 2015 ▸). Metal ions also serve as cofactors in many catalytic reactions. Some metal ions can induce water molecules to carry a partial charge that mediates certain catalytic reactions (Fife & Przystas, 1985 ▸), while metal ions with multiple prevalent oxidation states can achieve their catalytic role by changing oxidation state (Stadtman, 1990 ▸). There are also cases in which metal ions facilitate catalysis by maintaining the structure of the active site, thereby facilitating substrate binding (Solomon *et al.*, 2014 ▸; Pace & Weerapana, 2014 ▸).

Working with metal ions in macromolecular structures is a multidisciplinary problem that requires simultaneous consideration of chemical, crystallographic, biological and experimental aspects. While the chemical properties of the metal ions need to be addressed, modeling metal-binding sites in macromolecules also involves analysis of metal coordination chemistry and geometric distortions of the first coordination sphere that can be introduced by the macromolecule (Zheng *et al.*, 2008 ▸). In addition to chemical considerations, evaluation of metal-binding sites in experimentally determined structures needs to include crystallographic concerns such as the quality of the diffraction data (*e.g.* the resolution), as well as sample-preparation concerns such as nonspecific ion-binding sites introduced owing to a high concentration of metals in the sample. For example, the resolution of a macromolecular crystal structure is usually much lower than that of a small-molecule crystal structure; hence, the coordination bond length and bond angle are often observed with higher uncertainties (Zheng *et al.*, 2014 ▸). Last but not least, the structural and/or catalytic role of the metal in the biological process need to be accounted for during the modeling and validation process (Solomon *et al.*, 2014 ▸; Pace & Weerapana, 2014 ▸).

A survey shows that metal ions are modeled in ∼40% of all macromolecular structures deposited in the Protein Data Bank (PDB), yet the identification and accurate modeling of metals still pose significant challenges (Zheng *et al.*, 2014 ▸). The development of any tools for systematic analysis based on the protein structures in the PDB should take into account that these structural data are not error-free. Failure to consider this may result in inaccurate conclusions, as happened in a recent study of zinc coordination patterns (Yao *et al.*, 2015 ▸) that were shown to violate/ignore chemical and crystallographic knowledge (Raczynska *et al.*, 2016 ▸).

**Figure 1:**
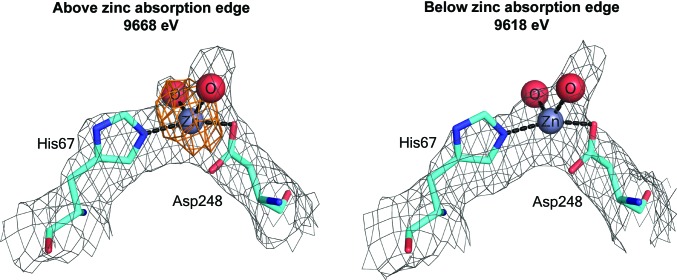
Zinc-binding site in serum albumin, determined using X-rays above and below the zinc absorption edge. Gray, 2*F*_o_ − *F*_c_; orange, *F*_o_ − *F*_c_.

In many cases, it is possible to unambiguously identify the type and/or location of metal ions by additional experiments (Garcia *et al.*, 2006 ▸). For example, the presence of a certain type of metallic element in the sample can be verified by inductively coupled plasma mass spectrometry (ICP-MS; Olesik *et al.*, 1998 ▸), while the local environment of metal-binding sites can be studied by both X-ray absorption near-edge structure (XANES) and extended X-ray absorption fine structure (EXAFS) techniques (Arcovito & della Longa, 2012 ▸; Hummer & Rompel, 2013 ▸). When the absorption edge of the target metal ion falls within the tunable wavelength range of the synchrotron station used for data collection, fluorescence scans and spectra can be employed to determine the presence of the particular metal in the crystal. Furthermore, the comparison of anomalous maps calculated from diffraction data collected at wavelengths above and below the absorption edge of a metal can enable the confident assignment of metals and their locations in the macromolecular structure (Ascone & Strange, 2009 ▸). For example, a recent paper describing circulatory zinc transport in albumins used this approach to unambiguously identify not only the major zinc-binding site on the two albumins but also several weaker zinc-binding sites (Handing *et al.*, 2016 ▸; Fig. 1 ▸).

In the absence of or in conjunction with anomalous diffraction data at the absorption peak of a metal, the type and location of metal ions can be inferred from the local binding environment in macromolecular structures. *CheckMyMetal* (*CMM*; http://www.csgid.org/csgid/metal_sites) is a validation algorithm that we have implemented for systematic inspection of the metal-binding architectures in macromolecular structures (Zheng *et al.*, 2014 ▸). The validation parameters that *CMM* examines cover the entire binding environment of the metal ion, including the position, charge and type of atoms and residues surrounding the metal. *CMM* can detect discrepancies from target values of the parameters that it assesses, and highlight potential problems in metal assignment and modeling. Hence, *CMM* is a convenient validation tool that is complementary to existing experimental methods for metal identification in macromolecular structures. As of 17 January 2017, *CMM* has validated 3801 structures from the PDB and 9558 structures uploaded by 2385 users from 47 countries. The validation algorithm in *CMM* initially published in 2014 (Zheng *et al.*, 2014 ▸) has been continuously mastered and re-evaluated as a result of numerous individual validation requests from *CMM* users. Here, we describe a selected set of practical examples to representatively illustrate the potential caveats and pitfalls that one might encounter when validating metal-binding sites in macromolecular structures.

## 2. Materials and methods   

*CMM* can diagnose all metal-binding sites in macromolecular structures with coordinates in the Protein Data Bank (PDB) format. It uses six experimental method-independent parameters and two X-ray crystallography-specific parameters to assess the quality of each modeled metal-binding site. The six experimental method-independent parameters evaluate (i) the atomic composition of the first coordination sphere of the metal (the *ligand* parameter; Zheng *et al.*, 2008 ▸; Harding *et al.*, 2010 ▸); (ii) the overall valence of the coordination bonds and completeness of the first coordination sphere according to the bond-valence model (the *valence* and *nVECSUM* parameters; Brown *et al.*, 2015 ▸; Müller *et al.*, 2003 ▸) and (iii) the geometric arrangement of the atoms coordinating the metal in the first coordination sphere (the *geometry*, *gRMSD* and *vacancy* parameters; Kuppuraj *et al.*, 2009 ▸). The two X-ray crystallo­graphy-specific parameters evaluate the consistency of the temperature factor and occupancy between the modeled metal and its binding environment (*occupancy* and *B-factor*).

*CMM* uses a red–amber–green (RAG) color scheme to denote each of the eight parameters as an outlier (red), borderline (amber) or acceptable (green) when applicable. The thresholds for classifying the coordination sphere composition (the *ligand* parameter) and geometries (the *geometry* parameter) into either outlier, borderline or acceptable zones are based on statistics described previously (Zheng *et al.*, 2008 ▸; Harding *et al.*, 2010 ▸; Kuppuraj *et al.*, 2009 ▸). The thresholds for classifying the other four experimental, method-independent parameters (the overall *valence*, *nVECSUM*, *gRMSD* and *vacancy* parameters) were empirically selected on the basis of the distributions of these parameters in a benchmark data set consisting of high-resolution (≤1.5 Å) metal-containing X-ray structures from the PDB (Zheng *et al.*, 2014 ▸). For the overall valence parameter, multiple distributions were used, subdivided both by metal identity and assumed oxidation state (valence): +1 (Na^+^/K^+^/Cu^+^), +2 (Mg^2+^/Ca^2+^/Mn^2+^/Fe^2+^/Co^2+^/Ni^2+^/Cu^2+^/Zn^2+^) or +3 (Fe^3+^/Co^3+^/Ni^3+^). For each metal, borderline and outlier zones are defined symmetrically both above and below the acceptable range for the corresponding valence. The borderline and outlier thresholds are >0.10 and >0.23, respectively, for *nVECSUM*; >13.5 and >21.5°, respectively, for *gRMSD*; and >10 and >25%, respectively, for the *vacancy* parameter. The thresholds for classifying the X-ray crystallography-specific parameters (the *occupancy* and *B-factor* parameters) were also empirically selected. For the occupancy parameter, only full occupancy is defined as acceptable, partial occupancy is borderline and essentially zero occupancy (≤0.01) is an outlier. The *B-factor* parameter shows two values: the *B* factor of the metal atom and the ‘environmental’ *B* factor, which is the bond-valence-weighted mean of the *B* factors of all ligand atoms. ‘Outlier’ indicates that the metal *B* factor is dissimilar to the ‘environmental’ *B* factor, while ‘acceptable’ indicates that metal *B* factor is similar to the ‘environmental’ *B* factor. A detailed description of and rationale for each of these eight parameters have been published in the *CMM* protocol (Zheng *et al.*, 2014 ▸).

## 3. Results and discussion   

The cases for modeling and validating various metals that we discuss here address the most commonly encountered issues in practical macromolecular crystallography. Our validation procedure focuses on mononuclear metal-binding sites. Metal clusters with two or more metal centers are handled as individual metal-binding sites; their validation as a whole cluster has not been parameterized by *CMM* and is beyond the scope of this paper. Here, we intend to use a case-study approach to describe the potential caveats in the validation of metal ions, with some examples illustrating the possible misconceptions of crystallographers and other biomedical researchers not necessarily versed in metal coordination chemistry. Since our examples are organized by the type of metal, *CMM* users may refer to the specific section for the practical issues that they may encounter during the modeling of that specific metal ion. The names of the eight validation parameters used in *CMM* (*ligand*, *valence*, *nVECSUM*, *geometry*, *gRMSD*, *vacancy*, *occupancy* and *B-factor*) will be italicized throughout this discussion.

### 3.1. Alkali and alkaline-earth metals   

Alkali and alkaline-earth metals usually play a structural role in stabilizing the fold of macromolecules or maintaining a certain conformation. Magnesium also plays a catalytic role in a variety of enzymes such as the T4 ligase (Cherepanov & de Vries, 2002 ▸) or ribozyme (Scott *et al.*, 1995 ▸). Alkali and alkaline-earth metals are also major contributors to charge compensation in highly acidic regions in macromolecules, especially for the polyphosphate backbone in nucleic acids (Owczarzy *et al.*, 2004 ▸; Zheng *et al.*, 2015 ▸; Várnai & Zakrzewska, 2004 ▸) or nucleotide-binding P-loop proteins (Porebski *et al.*, 2012 ▸; Via *et al.*, 2000 ▸). Alkali and alkaline-earth metal-binding sites in macromolecular structures are mostly six-coordinated with octahedral geometry (Kuppuraj *et al.*, 2009 ▸), yet the spatial arrangement of ligands around the metal poses an important restriction for metals with shorter metal–ligand distances. A rule of thumb is that with shorter metal–ligand distances (and hence tighter first coordination sphere ligands) there is less wriggle room in the first coordination sphere, resulting in less deviation from the ideal octahedral geometry. According to this trend, magnesium has the tightest first coordination sphere closest to ideal octahedral geometry, with a typical Mg—O distance of around 2.1 Å, followed by sodium and calcium with typical metal–oxygen distances of 2.4–2.5 Å, while K^+^ has the loosest first coordination sphere, with typical K—O distances of >2.7 Å. Potassium ions can accommodate distorted octahedral geometry without introducing clashes between coordinating ligands (Harding, 2002 ▸; Kim *et al.*, 2016 ▸). Alkali and alkaline-earth metals coordinated by more than six ligands are also found in the PDB. These cases are caused by the presence of bidentate ligands, with seven-coordinated metal sites involving one bidentate ligand and eight-coordinated metal sites involving two bidentate ligands (Zheng *et al.*, 2008 ▸).

Alkali and alkaline-earth metals are most frequently coordinated by acidic amino-acid side chains, and most alkaline-earth metals will bind to carboxyl side chains from Asp or Glu (Zheng *et al.*, 2008 ▸). Typically, a monovalent alkali metal (Na^+^ or K^+^) binding site is coordinated by zero or one carboxyl side chains, while a divalent alkaline-earth metal (Mg^2+^ or Ca^2+^) binding site is coordinated by at least two carboxyl side chains in the first coordination sphere (Harding, 2004 ▸). The presence of water molecules to complete the rest of the first coordination sphere is also crucial for the even distribution of charges into the local environment of the metal-binding site (Kim *et al.*, 2016 ▸). Moreover, water molecules in the first coordination sphere usually form hydrogen bonds to a carboxyl side chain in the second coordination sphere for additional charge neutralization, especially in cases when the first coordination sphere involves only water molecules (Harding *et al.*, 2010 ▸).

The presence of a hydroxyl O atom (from Ser or Thr) in the first coordination sphere is a much weaker determinant of binding-site specificity. However, for alkali metals, which only need a single unit of charge compensation, hydroxyl O atoms can also provide a small partial negative charge and an electron pair to form a coordination bond. Main-chain carbonyl O atoms are also good coordinating atoms for alkali metals, but not for alkaline-earth metals owing to their poor capacity for charge compensation. Indeed, the hydroxyl group of Ser/Thr and main-chain O atoms commonly coordinate sodium and potassium, but not magnesium and calcium (Zheng *et al.*, 2008 ▸). In nucleic acids, the phosphate moiety provides the most favorable interactions towards alkali and alkaline-earth metals, followed by carbonyl groups from the nucleobases, as exemplified by the investigation of magnesium-binding sites in nucleic acid crystal structures (Zheng *et al.*, 2015 ▸).

Similar features regarding the coordinating ligands have been observed in heavier alkali and alkaline-earth metals such as Rb^+^, Sr^2+^ and Ba^2+^, albeit with longer metal–ligand distances in coordination geometry (Kim *et al.*, 2016 ▸). Fortunately, the locations of heavier alkali and alkaline-earth metals sites are readily distinguishable by a much higher peak in the electron-density map when compared with Na^+^, Mg^2+^ and water molecules (Nayal & Di Cera, 1996 ▸). Hence, the accurate characterization of heavier alkali and alkaline-earth metals also depends on the presence of strong peaks in the electron-density map in addition to the characteristic coordinating geometry and ligands. Unfortunately, sometimes a low *B* factor is used as the sole criterion to assign magnesium or sodium, which does not take into account that these ions are isoelectronic with water. Using the *B*-factor criterion alone resulted in the placement of 1896 Mg atoms in just four structures (PDB entries 2a68, 2a69, 1smy and 1iw7; Dauter *et al.*, 2014 ▸).

#### 3.1.1. Sodium: differentiating it from magnesium and water   

**Figure 2:**
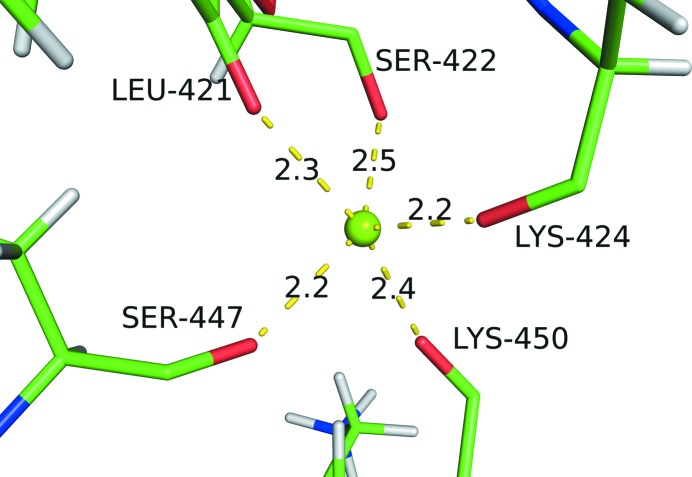
Mg998 in the CWP6 protein from the CWB2 cell-wall-anchoring module of the *C. difficile* cell-wall proteins CWP8 and CWP6

Sodium can be mistakenly modeled as either a water molecule or a magnesium ion even by experienced crystallo­graphers if only the experimental agreement between model and electron-density map is taken into consideration. In the crystal structures of the *Clostridium difficile* cell-wall proteins Cwp8 and Cwp6 (PDB entry 5j72; Usenik *et al.*, 2017 ▸), the metal ion coordinated by the main-chain O atoms of Leu421, Ser422, Lys424, Ser447, Lys450 was initially modeled as a magnesium ion because the metal–oxygen distances are close to the ideal Mg—O distance of 2.08 Å (Fig. 2 ▸). However, several chemical features indicate that the modeling of a sodium ion in the place of magnesium would result in a better fit. First of all, a magnesium ion carries more charge than a sodium ion and thus needs to be surrounded by more acidic residues such as Asp and Glu, while in this case all coordinating ligands are main-chain O atoms. Secondly, the first coordination sphere of magnesium is strictly octahedral, while sodium ions are less strict and can be five-coordinated, as observed in the model (Fig. 2 ▸). Last but not least, the overall *valence* favors a monovalent sodium (*valence* = 1.2) over a divalent magnesium (*valence* = 0.9) as determined by *CMM* (Table 1 ▸), although the individual distances (2.07–2.49 Å) fall between the ideal Mg—O distance (2.08 Å) and Na—O distance (2.41 Å). Upon *CMM* validation, a sodium ion is suggested as a better fit.

Distinguishing sodium ions from water molecules based on the distances to their ligands can be challenging because the sodium–oxygen distance (2.4–2.5 Å) overlaps with the distance of hydrogen bonds from water to its coordinating ligands (2.5–3.5 Å). Although sodium ions are typically six-coordinated with octahedral geometry, five-coordinated and four-coordinated sodium ions do sometimes exist in macromolecular structures, which can be confused with water molecules, which are typically four-coordinated with two hydrogen-bond donors and two hydrogen-bond acceptors in a tetrahedral coordination (Raschke, 2006 ▸). The bond-valence method (Nayal & Di Cera, 1996 ▸; Müller *et al.*, 2003 ▸), which has been incorporated as part of *CMM* (Zheng *et al.*, 2014 ▸), has been demonstrated to be effective at differentiating sodium ions from water molecules. If replacing a water molecule with a sodium ion results in an overall valence close to the unit of valence in *CMM* (*valence* between 0.7 and 1.3), that water molecule is likely misidentified and interpreting it as a sodium ion would be more chemically sensible. In some cases, sodium ions can be differentiated from water molecules by the type of coordinating atoms: metals cannot participate in hydrogen bonding, while water molecules are commonly coordinated by a hydrogen donor such as an amino group of the protein backbone or an amide group of Asn and Gln.

#### 3.1.2. Magnesium   

Magnesium is one of the most characteristic ions to identify because it possesses a compact and tight first coordination sphere with strict octahedral geometry and a typically short Mg—O distance of 2.08 Å. Owing to this compactness, small deviations from the ideal octahedral geometry would easily result in a clash in the first coordination sphere. Moreover, either under-coordinated or over-coordinated magnesium sites are rarely found. In addition to acidic coordinating ligands that compensate charge, the presence of water molecules in the first coordination sphere of a magnesium ion is especially important to fill all of the unoccupied vertices in the octahedral geometry. Naming the waters that coordinate magnesium ions with coordination numbers 1 to 6 (ligand three-letter codes MO1, MO2, MO3, MO4, MO5 and MO6) in macromolecular structures deposited in the PDB was common until it was abandoned during PDB remediation efforts (Henrick *et al.*, 2008 ▸). When compared with calcium-binding sites, acidic coordinating ligands are more likely to be present in the second coordination sphere of magnesium-binding sites owing to the limited space in the first coordination sphere (Zheng *et al.*, 2015 ▸).

Statistical analysis of magnesium–ligand interactions from the PDB indicates the presence of two major peaks in the distribution of Mg—O distances, one at 2.08 Å and the other at 2.18 Å, while the CSD shows only a single peak of Mg—O distances at 2.08 Å (Zheng *et al.*, 2015 ▸). Close examination of sites with Mg—O distances at 2.18 Å reveals the use of an over-restrained Mg—O distance in model refinement. The presence of an incorrect Mg—O distance restraint (2.18 Å) comes from the default values in the CCP4 library (ener_lib.cif) used by the macromolecule-refinement programs currently in use (Table 2 ▸). This misleading default Mg—O distance will hopefully be updated in the near future. The new generation of refinement programs should use bond-valence values of each metal-binding site as a restraint, in addition to the bond-length values for each coordination bond. One should also pay extra attention to the possible presence of incorrectly restrained Mg—O distances when examining existing magnesium-binding sites in the PDB.

The major coordinating ligands for magnesium are the same as other alkaline-earth metals: acidic residues such as carboxyl groups from Asp/Glu and phosphate groups from the nucleic acid backbone. Phosphates from ATP/ADP are also perfect coordinating ligands for magnesium and chelate magnesium in many biological processes (Zheng *et al.*, 2015 ▸). In addition to O atoms, endocyclic N atoms from an aromatic ring with *sp* ^2^ hybridization (—N=) are also found to coordinate mag­nesium, such as those in the imidazole ring from histidine side chains or a nucleobase. For example, a histidine side chain from the photosystem II chlorophyll-binding protein CP47 coordinates the magnesium from the bacteriochlorophyll molecule (Barber *et al.*, 2000 ▸). Exocyclic amino groups (—­NH_2_) are poorly suited to directly coordinate magnesium because they delocalize their lone electron pair into the heterocyclic ring and can coordinate metals only after deprotonation or a proton tautomeric shift, which can be induced by transition metals such as platinum or zinc but not by magnesium (Lippert, 2000 ▸). Endocyclic N atoms with *sp* ^3^ hybridization (—NH— and –N<) are also unfavorable to coordinate magnesium because the only lone electron pair is delocalized and is directed perpendicular to the aromatic ring.

#### 3.1.3. Potassium: differentiating it from chloride and water   

Potassium has the largest ionic radius among all commonly encountered metals in macromolecules, with typical K—O distances longer than 2.7 Å. The loose coordination sphere of potassium is unlikely to be confused with that of any other metal ion. However, there are good reasons why potassium may sometimes be modeled as chloride. First of all, the K^+^ cation and Cl^−^ anion both possess the same number of electrons and thus have the same diffraction power in the electron-density map. Moreover, the X-ray absorption *K* edges for potassium (3.43 Å) and chloride (4.39 Å) are similar and are both well above the wavelength accessible in standard anomalous diffraction experiments. Therefore, potassium cannot be differentiated from chloride based on the presence of a weak peak in the anomalous map alone. Secondly, both potassium and chloride commonly exist under physiological conditions at high concentrations. Thirdly, the K—O bond distance and the Cl—O bond distance are comparable, especially in macromolecular structures, where the resolution is usually not high enough to distinguish small nuances in bond distances. Lastly, unlike divalent cations, which usually have an acidic ligand(s) present in the first coordination sphere, the unit charge of either potassium or chloride can easily be distributed over the neutral coordinating ligands in the first coordination sphere. Overall, the many common features shared by both potassium- and chloride-binding sites require careful examination to distinguish between them.

Potassium can be differentiated from chloride by the type of coordinating atoms since potassium cannot participate in hydrogen bonding, while chloride is commonly coordinated by a hydrogen donor such as an amino group. Moreover, the typical geometry of potassium-binding sites is octahedral, while that of chloride-binding sites is tetrahedral. However, owing to limited resolution in macromolecular X-ray crystallography, coordinating ligands in the first coordination sphere of potassium are often incompletely modeled in macromolecular structures and therefore render potassium ions similar to chloride ions in terms of geometry. Additionally, potassium- and chloride-binding sites may be distinguished by evaluating the charge of the local environment. Acidic residues such as the carboxyl group in Asp/Glu or phosphate groups from the nucleic acid backbone should be in the first or second coordination sphere of potassium-binding sites. On the other hand, chloride-binding sites benefit from positively charged residues in the local environment, such as lysine or arginine in either the first or the second coordination sphere. It is also possible to distinguish potassium from chloride by the bond-valence method, such as by the use of calcium bond-valence (CBVS) values (Müller *et al.*, 2003 ▸). Potassium can be distinguished from chloride by *CMM* using the overall valence in cases with the complete first coordination sphere, although complete coordination spheres are rarely observed for these ions.

**Figure 3:**
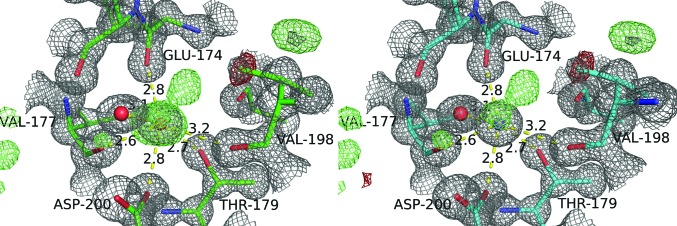
A water-binding site in the structure of proteinase K (PDB entry 3i34) which may be better interpreted as potassium.

Potassium may also be confused with water because the typical potassium–oxygen distance (2.7–3.2 Å) overlaps with the distance of hydrogen bonds from water to its coordinating ligands (2.5–3.5 Å). The similar principle about the octahedral geometry for alkaline metals and tetrahedral geometry for water applies to distinguish potassium from water, except that the K—O distance is longer than the Na—O distance. Additionally, the property that potassium possesses more electrons than either sodium or water can be effectively used to differentiate potassium from water. If a water molecule is modeled instead of a potassium ion, an unusually low *B* factor or a positive electron-density peak will be observed. For example, in the crystal of proteinase K at high resolution (PDB entry 3i34), water-binding site HOH311 may be better interpreted as potassium according to both the higher electron density and the octahedral geometry (Fig. 3 ▸).

#### 3.1.4. Calcium   

Calcium ions can stabilize the secondary and tertiary structures of many enzymes and are often critical for their function. For example, Ca^2+^ maintains the ordered structure of the copper-binding site in the case of nitrous oxide reductase (PDB entry 5i5m; Schneider & Einsle, 2016 ▸). Oxygen is the predominant ligand for calcium, while nitrogen is rarely observed to coordinate calcium (Zheng *et al.*, 2008 ▸). Historically, perhaps because of the lack of a metal-binding site validation tool, calcium was mistakenly modeled as magnesium in the crystal structure of the mature and fully active Der p 1 allergen (PDB entry 2as8; de Halleux *et al.*, 2006 ▸; Zheng *et al.*, 2008 ▸). The *valence* changed from 0.9 for magnesium to 1.8 for calcium (Table 1 ▸). The *valence* should be 2 for both of these divalent cations. Hopefully, such cases should become rather infrequent with the popular use of the *CMM* server (Zheng *et al.*, 2014 ▸).

From the perspective of geometry, calcium-binding sites share similar traits to those of sodium-binding sites because they both have octahedral geometry with a metal–oxygen coordination bond distance around 2.4–2.5 Å. Such similarity in coordinating geometry would render CBVS values (Müller *et al.*, 2003 ▸) an ineffective method of distinguishing between calcium- and sodium-binding sites. Fortunately, despite similar octahedral geometry and metal–oxygen distance, calcium-binding sites possess many characteristic features that sodium-binding sites lack, including both a higher number of electrons and a higher charge. Therefore, it is usually quite straightforward to distinguish calcium-binding sites from sodium-binding sites by looking at the electron-density maps, *B* factors and the presence of many carboxyl groups from Asp/Glu in the first coordination sphere. In addition, calcium can be detected by the presence of weak anomalous map peaks, especially if the diffraction data are collected at longer wavelengths, such as 1.5 Å or longer.

### 3.2. Aluminium fluoride   

Protein structures that contain metal fluorides MF_*x*_ as ligands that imitate a phosphoryl group or phosphate include AlF_4_^−^ in octahedral geometry, as well as AlF_3_ or MgF_3_^−^ in trigonal bipyramidal (TBP) geometry. AlF_4_^−^ mimics ‘in-line’ anionic transition states for phosphoryl transfer, while AlF_3_ and MgF_3_^−^ mimic the TBP stereochemistry of the transition state. Careful examination of the electron density allows the differentiation of AlF_3_ or MgF_3_^−^ from pentacoordinated phosphorane owing to the higher number of electrons in phosphorus. However, it is difficult to distinguish AlF_3_ from MgF_3_^−^ in crystal structures owing to both the identical geometry and the equivalent electron density between aluminium and magnesium. Even though many such sites are modeled as AlF_3_ in the PDB, careful consideration should be given towards their interpretation as MgF_3_^−^, especially when the crystallization condition is above pH 7.5, since Al^3+^ gradually precipitates in basic crystallization conditions. Enzymes with metal fluorides bound as ligands use Mg^2+^ as a catalytic metal, so Mg^2+^ is always present in the buffer and MgF_3_^−^ can take over AlF_3_-binding sites. The replacement of AlF_3_ by MgF_3_^−^ in crystal structures from the PDB has also been verified using ^19^F NMR as an orthogonal technique for a few structures, including protein kinase A on phosphoglycerate kinase, β-phospho­glucomutase (Jin *et al.*, 2014 ▸) and small G proteins (Jin *et al.*, 2016 ▸). For example, in the structure of a cAMP-dependent protein kinase (PDB entry 1l3r), one should consider that the crystallization condition is pH 8, and it is unlikely that AlF_3_ would be present in the structure owing to Al^3+^ precipitation (Madhusudan *et al.*, 2002 ▸). Therefore, replacing the AlF_3_ in this structure with MgF_3_^−^ would result in better agreement with known chemistry.

### 3.3. Transition metals in the fourth period   

Transition metals in the fourth period usually play a catalytic role in metalloenzymes. Transition metals participate in the catalytic process by (i) binding to substrates to orient them properly for reaction, (ii) mediating oxidation–reduction reactions through reversible changes in the metal-ion oxidation state or (iii) electrostatically stabilizing or shielding negative charges. The most commonly encountered transition-metal elements in macromolecular structures include Mn, Fe, Co, Ni, Cu and Zn. Some of the fourth-period transition metal cations with high redox activity can also be stable in multiple oxidation states, for example Fe^2+^ and Fe^3+^, Cu^2+^ and Cu^+^, Mn^3+^ and Mn^2+^, and Co^2+^ and Co^3+^.

Commonly observed ligands for transition metals in macromolecular structures include oxygen, nitrogen and sulfur. Transition metals can be coordinated by any nitrogen because it can induce the deprotonation or proton tautomeric shift even when there is no lone electron pair available. In addition to the side-chain carboxyls from Asp and Glu, side-chain N atoms from histidine and side-chain sulfurs from cysteine are both common ligands that coordinate most transition metals in the fourth period (Zheng *et al.*, 2008 ▸).

A previous survey of metal–ligand distances for these transition metals from the CSD shows that metal–oxygen distances are in the range 1.86–2.19 Å, while metal–nitrogen distances are in the range 1.67–2.29 Å (Table 3 ▸). Generally speaking, the distances between the fourth-period transition metal and its ligand are characteristically smaller than those between potassium/sodium/calcium and the corresponding ligand. However, Mg—O and Mg—N distances fall into the range of these distances. Some of these transition metals may fit into magnesium-binding sites in macromolecular structures or may replace magnesium in ADP/ATP-binding sites.

Besides the similarity in the type of ligands and metal–ligand distances, these transition metals in the fourth period also possess a similar number of electrons, making it difficult to differentiate between them just by the fit to the electron-density maps and by *B* factors. X-ray diffraction experiments performed below and above the absorption edge are the best way to differentiate between different types of transition metals. However, if all of the absorption edges of a metal are outside the accessible X-ray energy (wavelength) range, other characteristics of each of these transition metals, which are described in the following subsections, may be used to differentiate them.

#### 3.3.1. Manganese: similarity and difference to magnesium   

Although more than one transition metal in the fourth period may bind to magnesium-binding sites with various degrees of affinity, manganese (Mn^2+^) is the closest analogue to mag­nesium in biological systems (Bock *et al.*, 1999 ▸). Sometimes macromolecules with manganese replacing magnesium in the Mg-activated allosteric sites retain their biological activity, such as in the case of sarcoplasmic reticulum Ca-ATPase (González *et al.*, 1996 ▸). Manganese can also bind to ADP/ATP with similar effectiveness as magnesium to form Mn-ADP and Mn-ATP complexes and can be used by ATPase for energy processing (Huang *et al.*, 1995 ▸). Magnesium may also replace manganese effectively in many Mn-activated proteins (Spiro & Spiro, 1971 ▸).

Manganese and magnesium are exchangeable in many macromolecular structures because both of these divalent cations possess many common chemical properties including the coordination geometry, the type of coordinating ligands and the length of coordination bonds. Both manganese and magnesium exhibit tight octahedral geometry, with carboxyl O atoms and N atoms from histidine as the primary coordinating ligands (besides water) to complete the rest of the first coordination sphere. Although many Mg^2+^/Mn^2+^-binding sites share common characteristics, nuances in coordinating ligands exist. Manganese has a distinctive set of binding ligands compared with all other transition metals in the fourth period. While sulfur side chains from cysteine are a common coordinating ligand for most transition metals, manganese is an exception (Zheng *et al.*, 2008 ▸). Sites rich in histidine and other N atoms as coordinating ligands would favor manganese binding, while sites rich in carboxyl O atoms as coordinating ligands would favor magnesium binding. Despite the similarity, manganese can be effectively differentiated from magnesium in macromolecular structures by carefully examining the electron-density maps and the *B* factors, because each Mn^2+^ possesses 23 electrons, which is much greater than the ten electrons that each Mg^2+^ possesses. In addition, Mn^2+^ can be distinguished from Mg^2+^ by the presence of peaks in an anomalous difference map.

#### 3.3.2. Iron   

When compared with any of the other common metals discussed here, iron-binding sites in macromolecular structures from the PDB are rarely coordinated by water molecules (Zheng *et al.*, 2008 ▸). Iron-binding sites can be coordinated primarily by O atoms, such as in the cases of ferritin and transferrin. Yet other iron-binding sites are primarily chelated or coordinated by nitrogen in the first coordination sphere. For example, in the case of iron-containing heme as a complex, iron is coordinated by at least four N atoms in the iron–porphyrin plane. Heme in hemoglobin can bind molecular oxygen or a carbon monoxide molecule with a single O atom coordinated on top of the iron–porphyrin plane. Iron-binding sites with mostly nitrogen in the first coordination sphere may also have sulfur from either cysteine or methionine to complete the first coordination sphere if not nitrogen or oxygen. Iron-binding sites primarily coordinated by nitrogen usually exhibit an octahedral geometry if they are six-coordinated and a square-pyramidal geometry if they are five-coordinated. The typical iron–ligand distances in these sites are dependent on several factors including coordination number (five or six), oxidation state (II or III) and spin state (low spin or high spin). Consequently, it would be imprecise to define a single typical distance for a given iron–ligand coordination bond, especially in the case of iron–nitrogen interactions (Pidcock, 1995 ▸; Zheng *et al.*, 2017 ▸). For example, heme may display different iron–nitrogen distances depending not only on the oxidation state of the central iron but also on the spin state of the central iron (Zheng *et al.*, 2017 ▸). The iron–nitrogen distance could be as low as around 1.98 Å in the case of a low-spin Fe^2+^ ion or could be as high as around 2.24 Å in the case of a high-spin Fe^3+^ ion. Iron–oxygen distances are usually between 2.1 and 2.2 Å, while iron–sulfur distances are usually a little above 2.3 Å (Table 3 ▸).

**Figure 4:**
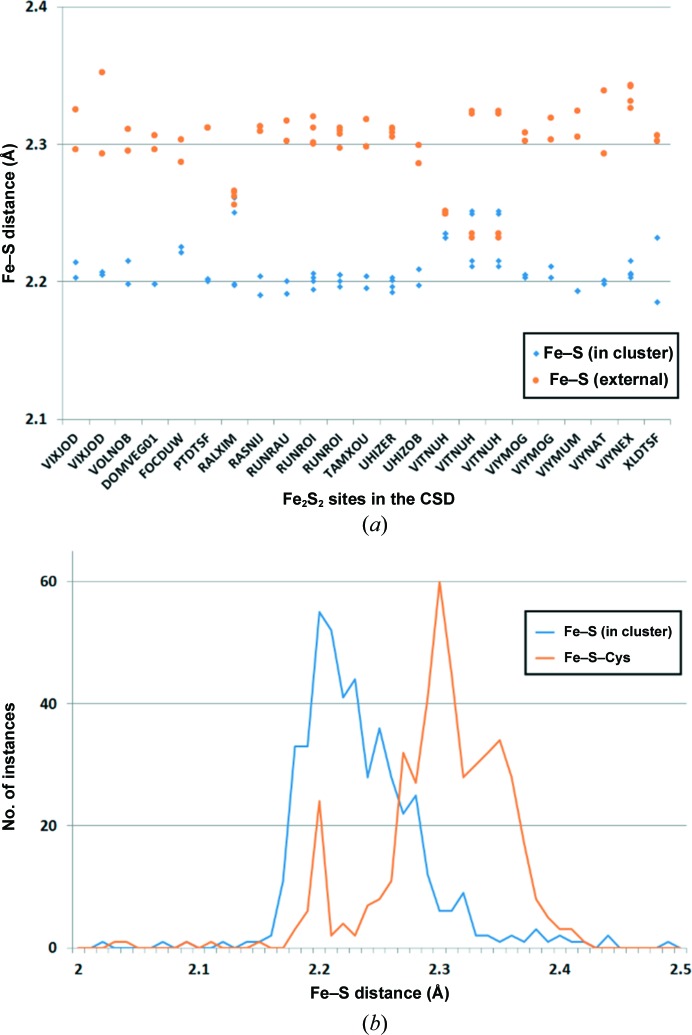
(*a*) Distribution of distances from iron to sulfur in an Fe_2_S_2_ cluster. For each Fe_2_S_2_ cluster (identified by CSD Refcode) the distances to all four sulfurs within the Fe_2_S_2_ cluster are shown in blue, while the distances to all four external sulfurs are shown in orange. (*b*) Distribution of Fe—S distances within Fe_2_S_2_ modeled in the PDB high-resolution data (<1.5 Å). Typical Fe—S distances are ∼2.2 Å between Fe and sulfurs within the Fe_2_S_2_ cluster, while typical Fe—S distances are ∼2.3 Å between Fe and external (cysteine) sulfurs.

In some cases, iron can also be coordinated exclusively by sulfurs. The iron–sulfur interaction is one of the most intriguing and complicated interactions of all metal–ligand interactions in macromolecular structures. Of all the abundant elements composing biological matter, iron is at the high end of the electronegativity spectrum for cations (1.83 according to the Pauling electronegativity scale), while sulfur is at the low end of the electronegativity spectrum for anions (2.58 according to the Pauling electronegativity scale). The small difference in electronegativity between iron and sulfur results in the presence of atypical coordination bonds when compared with the other metal–ligand interactions under investigation. This results in the formation of iron–sulfur clusters which are composed of two or more iron centers. The whole cluster is usually considered as ensembles of iron and sulfide centers in many circumstances. The most commonly observed clusters are Fe_2_S_2_, Fe_4_S_4_ and Fe_3_S_4_ clusters. In these clusters, each iron center is usually coordinated by four sulfur ligands; in the case of Fe_2_S_2_ with two internal sulfurs and two cysteines per iron, and in the case of Fe_4_S_4_/Fe_3_S_4_ with three internal sulfurs and one cysteine per iron. An exception to the sulfur-exclusiveness in iron–sulfur clusters is the cytochrome *b*_6_ *f* complex (PDB entry 4pv1), where the Fe_2_S_2_ cluster is coordinated by two histidines and two cysteines (Hasan *et al.*, 2014 ▸). Therefore, numerous complexes containing iron–sulfur interactions exhibit a four-coordinated geometry (tetrahedral or distorted tetrahedral), but not a six-coordinated or five-coordinated geometry as in the case of iron ions majorly coordinated by oxygen/nitrogen, resulting in an Fe—S distance of around 2.27–2.36 Å (Table 3 ▸). Further investigation of iron–sulfur binding sites in both the CSD and the PDB reveals that the majority of the cases exhibit an Fe—S distance distribution centered at ∼2.3 Å, with the single exception that the iron to internal sulfur distance is ∼2.2 Å in the case of Fe_2_S_2_ clusters (Fig. 4 ▸). This phenomenon is observed both in the CSD and in PDB data with a resolution higher than 1.5 Å. The reduction in Fe—S bond length is probably owing to the potential charge transfer in the local environment of the Fe_2_S_2_ cluster, allowing the sulfur within Fe_2_S_2_ clusters to carry a negative charge and cause a reduction in the Fe—S bond distance.

#### 3.3.3. Cobalt and nickel   

Cobalt and nickel are both similar to iron, but are less abundant in biological systems. From the X-ray macromolecular crystallography point of view, cobalt and nickel are nearly indistinguishable based on either the electron-density maps or the binding environment. An X-ray diffraction experiment performed both below and above the respective absorption edges is the best way to differentiate between these metals. Even from a chemical perspective, cobalt and nickel are similar to each other. A site that uses nickel as the metal center can use cobalt as well and still be functional, and *vice versa*. Cobalt may also replace iron in the porphyrin ring for oxygen transport (Yang & Huang, 2000 ▸). Proteins overexpressed for crystallographic studies commonly use a six-histidine tag in conjunction with immobilized metal-affinity chromatography (IMAC) resin using either nickel (*i.e.* Ni–NTA from Qiagen) or cobalt (*i.e.* TALON from Clontech) for purification, resulting in a nickel or a cobalt site coordinated by four histidine side chains. Despite the chemical similarity, proteins usually demonstrate a preference for either cobalt or nickel. For example, cobalt is well known to be part of vitamin B_12_ (Wuerges *et al.*, 2006 ▸) and nickel has been associated with vitamin C (Das & Büchner, 2007 ▸).

Both cobalt and nickel are usually six-coordinated with octahedral geometry, similar to an iron-binding site. In addition to nitrogen from aromatic rings such as histidine side chains, an amino group (—NH_2_) and ammonia (NH_3_) are ideal coordinating ligands for both cobalt and nickel. Similar to the tight magnesium–water complex, both cobalt and nickel can be coordinated only by either water or ammonia to form metal–water or metal–ammonia complexes such as cobalt hexamine(III). Similar to magnesium–water complexes, the cobalt–ammonia complex has the right size to fit in the grooves of various nucleic acid structures and may be used as a counter-ion to stabilize the acidic phosphate backbone in nucleic acid structures (Ramakrishnan *et al.*, 2003 ▸). If ammonia is available from the experiment, the presence of ammonia molecules in the first coordination sphere of a cobalt ion is important to fill all of the unoccupied vertices in the octahedral geometry. For example, in the crystal structure of ellipticine in complex with a 6 bp DNA (PDB entry 1z3f), cobalt hexamine(III) was modeled as a single cobalt ion, resulting in the modeling of a metal-binding site with poorly coordinated *geometry* upon *CMM* validation (Table 1 ▸). Interpreting the cobalt ions as six-ammonia-coordinated cobalt results in a better agreement of the model with the electron-density map. For Co^3+^ complexed with six ammonia molecules, the typical cobalt–ammonia distance is around 2.0 Å, while in cobalt–B_12_ complexes the typical cobalt–nitrogen distance is around 1.9 Å within the corrole plane. In the case of misidentified first coordination sphere, *CMM* will not be able to report the correct metal identity, yet it will assume that the modeled metal is correct and report the corresponding erroneous feature in the coordination sphere.

#### 3.3.4. Copper   

Besides nitrogen from histidine and sulfur from cysteine, the list of notable ligands that coordinate copper also includes sulfur from methionine (Zheng *et al.*, 2008 ▸). Preliminary analysis indicates that the distance between copper and S^γ^ from cysteine reported in the PDB peaks at around 2.2–2.3 Å, while the distance between copper and the S^δ^ atom of methionine peaks at around 2.5 Å.

Similar to cobalt and nickel, yet with a lower propensity, copper may form octahedral metal complexes with either water or ammonium (*e.g.* Cu·6H_2_O or Cu·6NH_3_) or a mixture of both (*e.g.* Cu·4NH_3_·2H_2_O). However, copper-binding sites in macromolecular structures are mostly four-coordinated with tetrahedral geometry, especially when coordinated by sulfur. For example, in the structure of the human copper chaperone ATOX1 (PDB entry 3iwx), a cisplatin molecule was modeled in the metal-binding site (Boal & Rosenzweig, 2009 ▸). *CMM* validation reveals that the ligands are too close to the metal (*valence* = 3.7) and the geometry is highly skewed (*gRMSD* = 24.1°) (Table 1 ▸). Interpretation of copper at the assigned cisplatin-binding site would result in a copper-binding site coordinated by four cysteines with Cu—S distances in the range 2.3–2.4 Å in a tetrahedral geometry, agreeing with the Cu—S distance that we published earlier (Table 3 ▸). The modeling of copper in the place of cisplatin at this site results in a better interpretation and has been discussed in detail previously (Shabalin *et al.*, 2015 ▸). If the diffraction data were available, analysis of the anomalous signal could have helped the interpretation.

#### 3.3.5. Zinc   

Unlike other transition metals, zinc lacks redox activity and is an ideal Lewis acid that polarizes water. These properties make it the most versatile catalytic metal widely used in many metalloenzymes, and its use spans all six enzyme classes (oxidoreductases, transferases, hydrolases, lyases, isomerases and ligases; Vallee & Galdes, 1984 ▸). Both structural and catalytic zinc sites are usually tetrahedral, although trigonal bipyramid cases exist, especially at catalytic sites in a less stable transition state (Yao *et al.*, 2015 ▸). The three major ligands are amino-acid side chains (carboxyl O atoms from aspartic acid and glutamic acid, N atoms from histidine and S atoms from cysteine), which coordinate zinc-binding sites in a wide variety of combinations. Zinc can be coordinated by only histidines (Avvaru *et al.*, 2010 ▸), only cysteines, a combination of both histidine and cysteine, a combination of histidine and carboxyl groups (Luo *et al.*, 2010 ▸) or a combination of histidine/cysteine/carboxyl groups. For example, a typical combination that facilitates fast ligand exchange is a zinc-binding site that contains one histidine, one cysteine and one carboxyl group in its first coordination sphere (Sousa *et al.*, 2007 ▸). This combination leads to a catalytic phenomenon known as carboxylate shift, which allows the zinc to switch from a monodentate coordination with a single O atom from a carboxyl group to a bidentate coordination using the both O atoms from the same carboxyl group. The carboxylate shift allows the zinc center to maintain a constant coordination number during catalysis (Sousa *et al.*, 2014 ▸).

In zinc-binding sites coordinated by only cysteine and histidine, the Zn–ligand distances and S—Zn—S angles are correlated with the number of cysteines and histidines (Touw *et al.*, 2016 ▸). Zn—S distances increase from 2.30 to 2.33 Å as the number of cysteines increases from one to four, and Zn—N distances also increase from 2.00 to 2.07 Å as the number of cysteines that coordinate the zinc increases from one to three. As the number of cysteines in the first coordination sphere increases from two to four, the S—Zn—S angles decrease from 116 to 109° (Touw *et al.*, 2016 ▸).

From the default values in the CCP4 library (ener_lib.cif) used by most popular macromolecule-refinement programs (Table 2 ▸), the Zn—N distance restraint (2.15 Å) would only be correct for six-coordinated zinc. Four-coordinated zinc should show a Zn—N distance between 2.00 and 2.07 Å (Touw *et al.*, 2016 ▸). Therefore, one should pay extra attention to the possible presence of incorrectly restrained Zn—N distances when examining zinc-binding sites in the PDB.

## 4. Conclusion   

The examples provided in this manuscript show that the identification and refinement of metal ions in macromolecular structures can be a challenging task even for experienced protein crystallographers. The assignment and validation procedure for metal ions requires the consideration of an array of chemical and biological information, such as the physiological function of the protein, the components of the crystallization solution, the pH value *etc*. The use of *CMM* as a validation tool guides researchers towards the examination of general attributes that include the type of ligand, geometry and metal–ligand distances, which can vary based on the valence contribution. It is difficult to create a general validation procedure that would cover all possible cases, but we believe that *CMM* is now the most advanced system to guide metal validation and alert the user to various potential pitfalls.

## Figures and Tables

**Table 1:** Summary of *CMM* results for the selected examples discussed in the text ‘Borderline’ parameters are italicized and underlined, and ‘outlier’ parameters are shown in bold and underlined.

Section	Case	ID	Res.	Metal	*Occupancy*	*B factor* (env.)	*Ligands*	*Valence*	*nVECSUM*	*Geometry*	*gRMSD* (°)	*Vacancy*	Bidentate
§[Sec sec3.1.1]3.1.1, Na, 5j72	Original	A:998	MG	Mg	1	11.5 (10.3)	O_5_	**0.9**	*0.15*	**Trigonal bipyramidal**	9.8	0	0
	Validated	A:998	NA	Na	1	11.5 (10.3)	O_5_	1.2	*0.15*	*Trigonal bipyramidal*	9.8	0	0
§[Sec sec3.1.4]3.1.4, Ca, 2as8	Original	A:1001	MG	Mg	1	*15.6 (27.4)*	O_6_	**0.9**	0.054	Octahedral	5.9	0	0
	Validated	A:1001	CA	Ca	1	25.5 (23.8)	O_6_	1.8	0.063	Octahedral	6.9	0	0
§[Sec sec3.3.3]3.3.3, Co, 1z3f	Original	B:31	CO	Co	1	*44.8 (27)*	**N_1_**	**0.16**	**1**	**Poorly coordinated**	N/A	N/A	0
	Validated	B:31	NCO	Co	1	*36.8 (31)*	N_6_	2.9	**0.24**	Octahedral	7.2	0	0
§[Sec sec3.3.4]3.3.4, Cu, 3iwx	Original	A:69	CPT	Pt1	*0.4*	37.2 (39.1)	N_2_S_4_	**3.7**	*0.22*	Octahedral	**24.1**	0	0
	Validated	A:400	CU	Cu	1	39.2 (36.7)	S_4_	1.8	0.048	Tetrahedral	6.9	0	0

**Table 2:** Selected examples that demonstrate the difference in metal–ligand coordination bond length between the default values in the CCP4 library (ener_lib.cif) and the statistical values from the CSD The coordination bond-length values from the CSD are presented for the most common geometries for that metal.

		Coordination bond length (Å)	
Metal	Ligand	Default value in CCP4 library (ener_lib.cif)	Derived from the bond-valence values from the CSD
Mg	N	2.09	2.19 (octahedral)
Mg	O	2.18	2.08 (octahedral)
Ca	O	2.32	2.34 (octahedral)
Zn	N	2.15	2.01 (tetrahedral)
Fe	N	1.98–2.09	1.98–2.24
Fe	O	2.04	2.11–2.17
Fe	S	2.30	2.33–2.35

**Table 3:** Metal–ligand distances in Å for fourth-period transition metals from the CSD (with standard deviations in parentheses) M represents metal, OC represents oxygen from carbon, OH_2_ represents oxygen from water, N represents nitrogen and S represents sulfur.

Metal	M—OC	M—OH_2_	M—N	M—S
Mn	1.91 (4), 2.19 (9)	2.19 (6)	1.99 (10), 2.29 (16)	2.36 (7), 2.64 (9)
Fe^2+^	2.18 (9)	2.10 (4)	1.97 (4), 2.18 (5)	2.27 (9)
Fe^3+^	2.04 (9)	2.10 (6)	1.67 (2), 2.08 (12)	2.28 (8)
Co	1.90 (2), 2.10 (9)	2.10 (5)	1.95 (5), 2.14 (6)	2.26 (11)
Ni	1.86 (4), 2.07 (7)	2.08 (6)	1.89 (4), 2.09 (7)	2.18 (3), 2.46 (10)
Cu^+^	2.10 (28)	1.98 (3), 2.33 (13)	2.02 (9)	2.34 (16)
Cu^2+^	2.12 (28)	1.97 (3), 2.37 (17)	2.03 (8)	2.33 (12)
Zn	2.15 (26)	2.09 (8)	2.10 (9)	2.38 (13)
